# Artifact reduction strategies for prosthetic heart valve CT imaging

**DOI:** 10.1007/s10554-012-0041-5

**Published:** 2012-04-05

**Authors:** Jesse Habets, Petr Symersky, Tim Leiner, Bas A. J. M. de Mol, Willem P. Th. M. Mali, Ricardo P. J. Budde

**Affiliations:** 1 Department of Radiology, University Medical Center Utrecht, P.O. Box 85500, E01.132, 3508 GA Utrecht, The Netherlands; 2 Department of Cardiothoracic Surgery, Academic Medical Center, Amsterdam, The Netherlands; 3 Department of Radiology, Gelre Hospital, Apeldoorn, The Netherlands

**Keywords:** Computed tomography, Prosthetic heart valve, Metal artifact reduction, Iterative reconstruction, Tube voltage

## Abstract

Multislice CT evaluation of prosthetic heart valves (PHV) is limited by PHV-related artifacts. We assessed the influence of different kV settings, a metal artifact reduction filter (MARF) and an iterative reconstruction algorithm (IR) on PHV-induced artifacts in an in vitro model. A Medtronic-Hall tilting disc and St Jude bileafet PHV were imaged using a 64-slice scanner with 100 kV/165 mAs, 120 kV/100 mAs, 140 kV/67 mAs at an equal CTDI_vol_. Images were reconstructed with (1) filtered back projection (FBP), (2) IR, (3) MARF and (4) MARF and IR. Hypo- and hyperdense artifacts volumes (mean mm^3^ ± SD) were quantified with 2 thresholds (≤−50 and ≥175 Hounsfield Units). Image noise was measured and the presence of secondary artifacts was scored by 2 observers independently. Mean hypodense artifacts for the Medtronic-Hall/St Jude valve (FBP) were 966 ± 23/1,738 ± 21 at 100 kV, 610 ± 13/991 ± 12 at 120 kV, and 420 ± 9/634 ± 9 at 140 kV. Compared to FBP, hypodense artifact reductions for IR were 9/8 %, 10/7 % and 12/6 % respectively, for MARF 92 %/84 %, 89/81 % and 86/77 % respectively; for MARF + IR 94/85 %, 92/82 %, and 90/79 % respectively. Mean hyperdense artifacts for the Medtronic-Hall/St Jude valve were 5,530 ± 48/6,940 ± 70 at 100 kV, 5,120 ± 42/6,250 ± 53 at 120 kV, and 5,011 ± 52/6,000 ± 0 at 140 kV. Reductions for IR were 2/2 %, 2/3 % and 3/4 % respectively, for MARF were 9/30 %, 0/25 %, 5/22 % respectively, MARF + IR 12/32 %, 4/27 % and 7/25 % respectively. Secondary artifacts were found in all MARF images. Image noise was reduced in the IR images. In vitro PHV-related artifacts can be reduced by increasing kV despite maintaining identical CTDI_vol_. Although MARF is more effective than IR, it induces secondary artifacts.

## Introduction

Prosthetic heart valve (PHV) assessment is a promising new application for multislice CT (MSCT) [1–7]. Although echocardiography is the mainstay of functional evaluation of prosthetic valves, it is hampered by acoustic shadowing and it may not be able to identify periprosthetic obstructive masses or false aneurysms [5–7]. The visualization of areas considered “off-limits” to echocardiography with MSCT allows detection of obstructive masses and may aid in the management of these patients [2, 5]. In addition, MSCT allows the detection of periprosthetic leaks, vegetations and degenerative changes in biological prosthetic valves and allows evaluation of leaflet motion in mechanical valves [1–7]. Despite the excellent spatial and good temporal resolution of current MSCT technology, PHV CT images vary in quality. For mechanical PHV, a variable amount of valve-induced artifacts remains due to the radiopaque and metal components of the PHV [2, 3, 8, 9]. Compounding the problem of artifacts, are the differences in PHV composition. PHV consisting of cobalt chromium components, such as the Björk-Shiley and Sorin tilting disc valves, are associated with severe artifacts that prohibit CT assessment of these valves. In contrast, modern mechanical PHV that consist of tungsten impregnated carbon leaflets and titanium or nickel alloy rings induce far less artifacts and allow a much more complete visualization of the periprosthetic anatomy [1, 2, 4, 6]. Because the masses interfering with normal PHV function and periprosthetic leaks are directly adjacent to the high attenuation components of the PHV, further reduction of the PHV-related artifacts may further improve the diagnostic yield of MSCT.

The problem of metal artifacts is ubiquitous in CT imaging and many different strategies have been devised to improve the image quality around metal objects. These strategies reflect the multiple mechanisms that cause these artifacts. On the one hand physics-related interactions such as beam hardening, scatter and photon starvation are important. On the other hand, algorithms used for reconstruction of these faulty raw data may augment artifacts by, for example, creating windmill artifacts and artifacts related to helical interpolation [9–12]. As mentioned above, these interactions are further complicated by the differences in PHV composition which have been related to the severity of artifacts. Hence, a single intervention, such as the increase of beam energy or iterative image reconstruction, may decrease some artifacts but not sufficiently eliminate them [10, 11].

Our goal in this study was to evaluate the effectiveness of three ways to reduce PHV-related artifacts: (1) variation of tube voltage (beam energy), (2) applying a metal artifact reduction filter, and (3) iterative image reconstruction. By comparing these three approaches for the reduction of PHV-related artifacts in an in vitro model, we sought to determine the effectiveness of each method for optimizing the MSCT image quality of PHV.

## Methods

### Valves

**Fig. 1:**
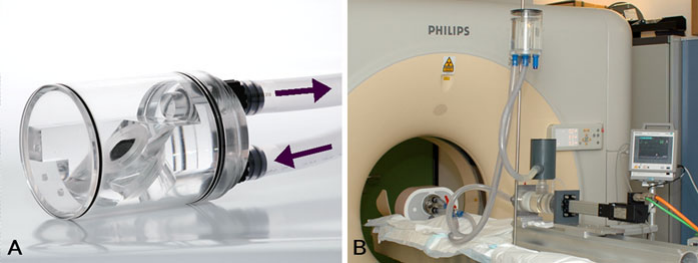
Polymethyl methacrylate (PMMA) perfusion chamber (**a**). Pulsatile in vitro model in 64 slice CT scanner (Brilliance 64, Philips Medical Systems, Cleveland, Ohio, USA) (**b**). (Reprinted with permission [9], Copyright ICR Publishers)

The valves were mounted in a previously described polymethyl methacrylate (PMMA) valve chamber [9] which was placed in a commercially available thoracic phantom (QRM GmbH, Möhrendorf, Germany, Fig. 1). The chamber was filled with water and the valve was positioned at a 45 degree angle to the scanner gantry simulating the approximate position of the aortic valve in vivo. No valve or leaflet motion was present. Two different PHVs were imaged in a fixed open position: (1) 27 mm St Jude masters bileaflet (St Jude Medical Inc., St Paul, MN, USA) and (2) 27 mm Medtronic Hall tilting disc (Medtronic Inc., Minneapolis, MN, USA).

### Scan protocol

All scans were performed on a 64-slice CT scanner (Brilliance 64, Philips Medical Systems, Cleveland, Ohio, USA). Because the metal artifact reduction filter can not yet be used with ECG-gated scans protocols, we adapted a standard scan protocol for thoracic imaging to have the same imaging parameters as a standard retrospectively gated CT of the heart. This was done by adjusting the following parameters: pitch 0.2, collimation 64 × 0.625 mm, 120 kV, matrix size 512 × 512, gantry rotation time 420 ms. Because a non-ECG-gated image acquisition results in much lower noise levels, we adjusted the mAs setting to a lower value at which the same level of noise (defined as the standard deviation (SD) of CT attenuation) was present as that found in earlier ECG-gated in vitro experiments [9] using a standard ECG-gated cardiac protocol. Noise was measured using a circular region of interest (diameter 1 cm) in a homogenous part of the PMMA structure of the valve chamber not affected by the PHV-related artifacts. For the adapted thoracic protocol at 120 kV, a mAs setting of 100 resulted in equal image noise.

For the experiment, three different acquisition protocols with different tube voltage settings with identical CTDI_vol_ and DLP values were used. For this, the adapted thoracic protocol was adjusted to 100 and 140 kV to yield identical CTDI_vol_ and DLP values obtained for the 120 kV, 100 mAs scan. This resulted in scans performed at 140 kV, 67 mAs and 100 kV with 165 mAs. Ten scans of each valve were performed with each kV, mAs setting. A standard reconstruction filter was used because the detailed cardiac filter is not available for non-gated scans. Images were reconstructed at 0.9 mm thickness with a 0.45 mm increment.

### Image reconstruction

Images were reconstructed in four different ways: (1) standard filtered back projection (FBP), (2) using iterative image reconstruction (IR, iDose, Philips Healthcare, Best, the Netherlands), (3) FBP combined with a metal artifact reduction filter (MARF, Philips Medical Systems, Best, the Netherlands), and (4) IR combined with MARF. According to the manufacturer the IR used in this study uses several cycles of iterative reconstruction and applies a maximum likelihood denoising algorithm based on Poisson statistics on the raw projection data. Subsequently the reconstructed images are compared to optimal anatomical structures in image space, allowing noise reduction without altering the characteristics and overall appearance of the initial image. Different levels of IR are possible with increasing influence on image reconstruction. The level used in this study leads to image denoising by 50 % according to manufacturer data.

MARF is an image-based algorithm which is based on interpolation of data for the reduction of artifacts. Importantly, the MARF algorithm uses the images provided by the reconstruction of the raw data with either FBP or IR.

### Image analysis

All image sets were transferred to a dedicated workstation for analysis (Extended Brilliance Workstation, Philips Medical Systems, Best, the Netherlands). High and low-density artifact volumes were quantified using two thresholds based on the densities of the surrounding structures according to a methodology described previously [9]. We chose threshold values that were approximately 3 SD above the Hounsfield Unit (HU) of PMMA measurement and below the HU of water, respectively. The chosen thresholds were ≥175 HU for hyperdense artifacts and ≤−50 HU for hypodense artifacts. Because the PHVs are composed of various radiopaque components such as a titanium ring and tungsten impregnated leaflets [9], the ≥175 HU threshold included the radiopaque components of the PHV as well. Hence, the percentage change in hyperdense artifacts measured with the ≥175 HU threshold underestimates the actual change in artifacts because the radiopaque components are included as well. Areas outside the valve chamber and other unrelated sources of artifacts were manually digitally excised in an identical manner for all scans.

In addition to the quantification of artifacts, all images reconstructed with the four different reconstruction algorithms were evaluated for possible induced changes in periprosthetic densities of the water and the PMMA contours. These secondary artifacts were considered induced if: (1) they were localized elsewhere or beyond primary artifact distribution in comparison with the normal FBP reconstruction; and (2) did not follow known contours of water and the PMMA structure. These induced changes were scored (present or absent) by two observers independently (JH and PS).

Image noise (defined as the SD of CT attenuation) was measured in all images using a circular region of interest (diameter 1 cm) that was placed in a homogenous part of the PMMA structure of the valve chamber not affected by the PHV-related artifacts.

### Data analysis

Data were analyzed using SPSS software (SPSS Statistics Version 15.0, SPSS Inc, Chicago, IL) and were presented as means ± SD. For each PHV type, a two-way repeated measures analysis was performed with reconstruction algorithm (FBP, IR, FBP + MARF, and IR + MARF) as within-subjects factor and tube voltage/scan protocol (100 kV, 165 mAs; 120 kV, 100 mAs and 140 kV, 67 mAs) as between-subjects factor, and hypo- and hyperdense artifacts as dependent variable. Because of a significant interaction between tube voltage and reconstruction algorithm, additional analyses were performed to analyze the main effects of tube voltage (one-way ANOVA) and reconstruction algorithm (repeated measures analysis).

For each PHV, a repeated measures analysis was performed with reconstruction algorithm as within-subjects factor and tube voltage/scan protocol as between-subjects factor and image noise as dependent variable. In case of violation of the sphericity assumption, Greenhouse-Geisser correction was applied. Post hoc pairwise testing with Bonferroni correction was performed to compare the different levels of the reconstruction algorithms and tube voltages. Statistical significance was defined as a *p* value <0.05.

## Results

### Radiation exposure

Scan length was equal for all scans at 109.8 mm. The CTDI_vol_/DLP values were exactly equal for all scans at 5.90 mGy and 960.4 mGy cm, respectively.

### Artifact volumes

Mean hypo- and hyperdense artifact volumes for different tube voltages are shown in Table 1 and 2. The reductions related to tube voltage and reconstruction algorithm were not proportional to each other e.g. a significant interaction was present for both hypo- and hyperdense artifacts for Medtronic Hall PHV (*F* value 2,914, *p* < 0.001 and 76, *p* < 0.001, respectively); and for St Jude bileaflet PHV (*F* value 12,502, *p* < 0.001 and 715, *p* < 0.001, respectively). Because of this interaction, the averages could not be compared as a group in the repeated measures analyses, but a *p* value was generated for each of the three kV settings and each reconstruction algorithm.

**Table 1:** The influence of tube voltage and reconstruction algorithm on hypodense PHV artifacts

PHV type	Scan protocol	FBP^a^	IR^a^	FBP + MARF^a^	IR + MARF^a^
Medtronic Hall tilting disc^b^	100 kV, 165 mAs	966 ± 23	875 ± 20 (−9 %)	81 ± 3 (−92 %)	56 ± 3 (−94 %)
	120 kV, 100 mAs	610 ± 13	548 ± 13 (−10 %)	69 ± 3 (−89 %)	48 ± 3 (−92 %)
	140 kV, 65 mAs	420 ± 9	371 ± 10 (−12 %)	57 ± 5 (−86 %)	40 ± 2 (−90 %)
St Jude Bileaflet^b^	100 kV, 165 mAs	1,738 ± 21	1,606 ± 20 (−8 %)	278 ± 3 (−84 %)	257 ± 3 (−85 %)
	120 kV, 100 mAs	991 ± 12	922 ± 8 (−7 %)	191 ± 4 (−81 %)	175 ± 2 (−82 %)
	140 kV, 65 mAs	634 ± 9	595 ± 6 (−6 %)	146 ± 3 (−77 %)	133 ± 2 (−79 %)

Between brackets percentage PHV artifact reduction compared to standard FBP

^a^Different reconstruction algorithms: Filtered Back Projection (FBP), Metal Artifact Reduction Filter (MARF), Iterative Reconstruction (IR)

^b^Manufacturer details: Medtronic Hall tilting disc (Medtronic, Minneapolis, MN, USA) and St Jude bileaflet (St Jude Medical, St Paul, MN, USA)

**Table 2:** The influence of tube voltage and reconstruction algorithm on hyperdense PHV artifacts

PHV type	Scan protocol	FBP^a^	IR^a^	FBP + MF^a^	IR + MF^a^
Medtronic Hall tilting disc^b^	100 kV, 165 mAs	5,530 ± 48	5,420 ± 42 (−2 %)	5,009 ± 32 (−9 %)	4,850 ± 26 (−12 %)
	120 kV, 100 mAs	5,120 ± 42	4,997 ± 7 (−2 %)	5,110 ± 32 (−0 %)	4,901 ± 18 (−4 %)
	140 kV, 65 mAs	5,011 ± 52	4,849 ± 26 (−3 %)	4,767 ± 135 (−5 %)	4,641 ± 89 (−7 %)
St Jude Bileaflet^b^	100 kV, 165 mAs	6,940 ± 70	6,770 ± 48 (−2 %)	4,829 ± 30 (−30 %)	4,733 ± 25 (−32 %)
	120 kV, 100 mAs	6,250 ± 53	6,070 ± 48 (−3 %)	4,663 ± 19 (−25 %)	4,541 ± 19 (−27 %)
	140 kV, 65 mAs	6,000 ± 0	5,740 ± 52 (−4 %)	4,708 ± 14 (−22 %)	4,523 ± 14 (−25 %)

Between brackets percentage PHV artifact reduction compared to standard FBP

^a^Different reconstruction algorithms: Filtered Back Projection (FBP), Metal Artifact Reduction Filter (MARF), Iterative Reconstruction (IR)

^b^Manufacturer details: Medtronic Hall tilting disc (Medtronic, Minneapolis, MN, USA) and St Jude bileaflet (St Jude Medical, St Paul, MN, USA)

### Influence of tube voltage on PHV artifact volumes

**Fig. 2:**
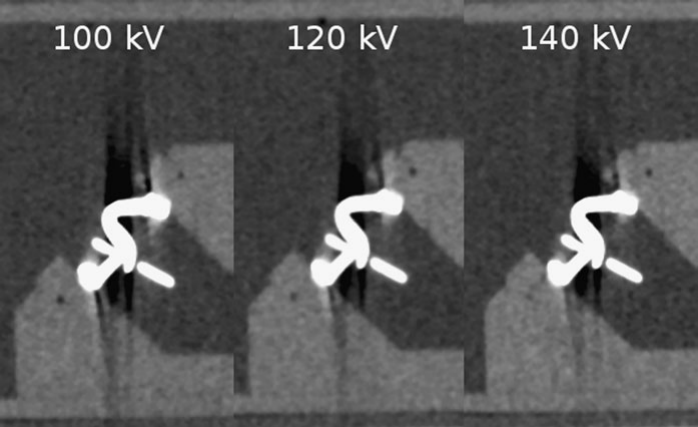
CT image reconstructions of Medtronic Hall tilting disc (Medtronic Inc., Minneapolis, MN, USA) reconstructed with filtered back projection and scanned with 100 kV (**a**), 120 kV (**b**) and 140 kV (**c**). Note the moderate reduction of hypodense artifacts and a slight reduction of hyperdense artifacts

For the Medtronic Hall tilting disc PHV, mean hypo- and hyperdense artifacts were significantly lower at 140 kV compared to 100 and 120 kV for all different reconstruction algorithms (All *p* values <0.001) (Tables 1, 2; Fig. 2). However for hyperdense artifacts, there was no significant difference between 100 and 120 kV when reconstructed with MARF + IR (*p* = 0.136).

For St Jude Bileaflet, mean hypo- and hyperdense artifacts were significantly lower in 140 kV scans compared to 100 and 120 kV scans for all different reconstruction algorithms (All *p* values <0.001) (Tables 1, 2). For hyperdense artifacts, there was no significant difference between 120 and 140 kV when reconstructed with MARF + IR (*p* = 0.154).

### Influence of reconstruction algorithm on PHV artifact volumes

For Medtronic Hall tilting disc PHV, mean hypo- and hyperdense artifacts were significantly different between the different reconstruction algorithms for each kV setting (All *p* values <0.001) (Tables 1, 2). No difference existed for hyperdense artifacts between FBP and MARF at 120 kV (*p* = 1.0), and between IR and MARF at 140 kV (*p* = 0.588).

For St Jude bileaflet PHV, mean hypo- and hyperdense artifacts were significantly different between the different reconstruction algorithm at each kV setting (all *p* values < 0.001) (Tables 1, 2). For both hypodense and hyperdense artifacts, the reduction were more pronounced when with MARF and MARF + IR compared to FBP and IR alone (Table 1, 2).

### Image noise

For each PHV, mean image noise is presented for different tube voltages and different reconstruction algorithms in Table 3. For both St Jude and Medtronic Hall PHV, mean image noise was not significantly different for different tube voltages (*p* = 0.133, *p* = 0.815, respectively). Image noise was significantly lower in scans reconstructed with IR alone and IR + MARF for both PHVs (*p* < 0.001).

**Table 3:** Mean image noise (±SD) in different reconstruction algorithms and scan protocols

PHV type	Scan protocol	Image noise (FBP^a^)	Image noise (IR^a^)	Image noise (FBP + MARF^a^)	Image noise (IR + MARF^a^)
Medtronic Hall tilting disc^b^	100 kV, 165 mAs	13.0 ± 1.3	10.2 ± 1.2	12.7 ± 1.3	10.5 ± 1.0
	120 kV, 100 mAs	12.1 ± 1.3	9.4 ± 1.2	12.4 ± 1.1	9.4 ± 1.2
	140 kV, 65 mAs	12.8 ± 1.4	9.7 ± 0.7	12.7 ± 1.1	9.7 ± 0.9
St Jude Bileaflet^b^	100 kV, 165 mAs	10.8 ± 0.5	8.6 ± 0.5	10.8 ± 0.6	8.4 ± 0.6
	120 kV, 100 mAs	10.7 ± 1.0	8.7 ± 1.2	10.8 ± 1.2	8.3 ± 1.0
	140 kV, 65 mAs	11.1 ± 0.9	8.6 ± 0.9	11.0 ± 1.1	8.7 ± 1.1

^a^Different reconstruction algorithms: Filtered Back Projection (FBP), Metal Artifact Reduction Filter (MARF), Iterative Reconstruction (IR)

^b^Manufacturer details: Medtronic Hall tilting disc (Medtronic, Minneapolis, MN, USA) and St Jude bileaflet (St Jude Medical, St Paul, MN, USA)

### Interpolation artifacts induced by MARF

**Fig. 3:**
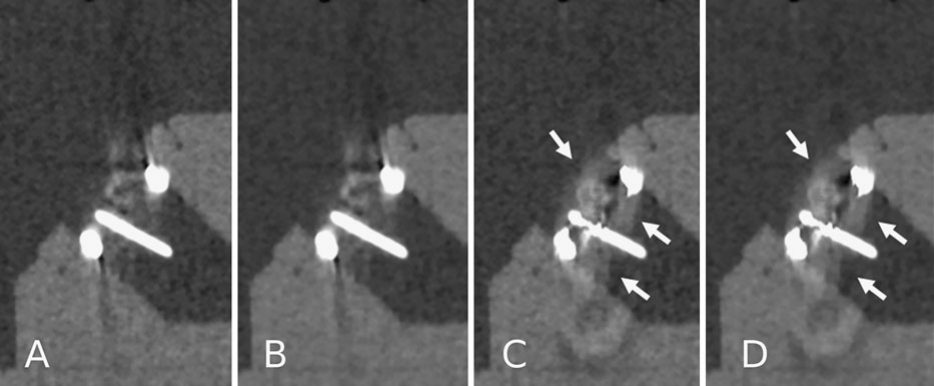
CT image reconstructions of Medtronic Hall tilting disc (Medtronic Inc., Minneapolis, MN, USA) with filtered back projection (**a**), iterative reconstruction (**b**), metal artifact reduction filter (**c**) and metal artifact reduction filter and iterative reconstruction (**d**). Note the secondary artifacts present in the MARF reconstructions (*arrows*) (**c**, **d**). These artifacts are not present in the other reconstructions FBP (**a**) and IR (**b**)

**Fig. 4:**
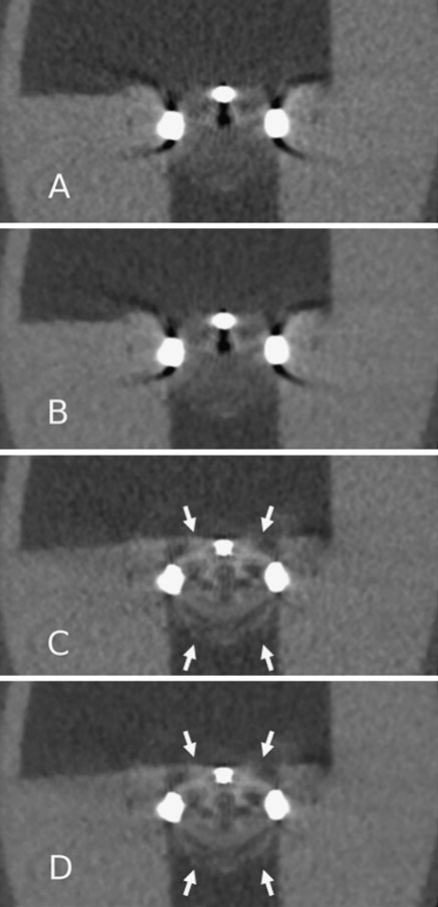
CT image reconstructions of Medtronic Hall tilting disc (Medtronic Inc., Minneapolis, MN, USA) with filtered back projection (**a**), iterative reconstruction (**b**), metal artifact reduction filter (**c**) and metal artifact reduction filter and iterative reconstruction (**d**). Note the secondary artifacts present in the MARF reconstructions (*arrows*) (**c**, **d**)

For both St Jude bileaflet PHV and Medtronic Hall tilting disc PHV, MARF and MARF + IR caused interpolation artifacts in each of the 10 scans (Figs. 3, 4). In the scans reconstructed with FBP and IR alone no interpolation artifacts were present.

## Discussion

The principal findings of this study are: (1) increasing tube voltage (kV) while maintaining identical CTDI_vol_ reduced mainly hypodense and to a lesser extent hyperdense PHV artifacts; (2) IR reduced PHV artifacts to a lesser extent than an increase in kV but reduced image noise compared to scans reconstructed with FBP; and (3) although MARF is very effective in reducing hypodense artifacts, it induced artifacts and deformed the periprosthetic anatomy.

For MSCT imaging of PHV using commercially available technology, high energy photons may be most effective for the reduction of artifacts. Although the effect of iterative reconstruction seems to be little with respect to artifact reduction, it does reduce image noise which may enhance image quality.

Several mechanisms of PHV-related artifacts have been proposed. For one part, the artifacts are related to radiopaque components. All currently implanted mechanical PHVs have tungsten impregnated leaflets and prosthetic rings consisting of metal alloys. These metal alloys vary from titanium, nickel to cobalt-chrome alloys and are associated with various gradations in artifact severity [1, 4, 8, 9]. The artifacts caused by these metal alloys form the rationale for the use of a metal artifact reduction filter. On the other hand, motion of the PHV due to the annular motion in concert with cardiac contractions and leaflet motion are another source of artifacts. For example, the opening and closing motion of leaflets has been associated with increases in hyper- and hypodense artifacts [8]. In clinical acquisitions, annular motion also may increase hypo- and hyperdense PHV artifacts. In this study, we aimed to evaluate different strategies to reduce the artifacts associated with the metal and radiopaque components of PHVs.

Increasing photon energy reduced photon starvation as a cause of PHV-related artifacts. IR demonstrated an additional hypo- and hyperdense artifact reduction depending on tube voltage level (6–12 % and 2–4 %, respectively). Interestingly, the reduction of tube current did not cause any measurable increase in image noise when measured in the PMMA structure surrounding the PHV [13]. This may have important implications for clinical scanning of PHV: standard coronary protocols should be modified by increasing tube voltage while maintaining equal CTDI_vol_ (reducing mAs) to reduce PHV-related artifacts. Although 140 kV has been employed clinically to enhance visibility of bioprostheses, it was done without reduction of tube current and at the expense of an increased radiation exposure [14]. Our results demonstrate that with an equal dose, the increased tube voltage may represent a readily available and reproducible way to enhance PHV image quality because of PHV artifact reduction. Increased tube voltage results in clinically relevant hypodense PHV artifact reductions (Table 1). Although the reductions in hyperdense artifacts are relatively small, the volume of the radiopaque components is included in the ≥175 HU threshold. When corrected for these components (i.e. tungsten impregnated leaflets and metal alloy prosthetic ring [8]) the artifact reductions amount to 15 and 18 % for the Medtronic Hall and St Jude valve, respectively.

Another source of artifacts related to metal objects is scatter and noise [15]. Commercially available iterative reconstruction algorithms are effective in denoising images and improve image quality based on Poisson statistics [11, 15]. Our results confirm that IR addresses another mechanism of PHV-related artifacts (it reduces image noise) and has some incremental value to the increase in tube voltage. The decrease in hypodense artifact volume may be attributed to the reduction of image noise compared to the scans reconstructed with FBP. However, a higher tube voltage is more effective for PHV artifact reduction. This approach is similar to the strategy of Boas and Fleischmann [15] who specifically aimed at reducing several mechanisms of metal artifacts. Their findings may not be completely comparable to ours, because of the difference in size and in composition of PHVs. Clinically, IR may be used next to modified exposure parameters to optimize PHV image quality and may possibly reduce radiation dose. Previous in vitro work demonstrated that iterative reconstruction enables a 50 % dose reduction (120 kV, 300 mAs) compared to a standard dose (120 kV, 600 mAs) with FBP without an increase in image noise or PHV-related artifacts [16].

The MARF algorithms have been generally conceived to reduce the artifacts caused by large metallic objects such as hip prostheses [17]. In fact, due to the variation in composition and size of various metal objects, MARF may either be effective in restoring image quality or detrimental by inducing artifacts. Despite advances, a recurrent problem with MARF is the interpolation of detector values considered altered by the metal. Nothing is “recovered” by interpolation but an average value of the surrounding voxels is assumed to be appropriate [18, 19]. Hence interpolation artifacts in both PHVs in all scans remain a limitation of this technique as demonstrated by our results (Figs. 3, 4). In clinical practice, these interpolation artifacts are situated on the location where PHV pathology can be expected. These interpolation artifacts may hamper accurate diagnostic assessment of PHV pathology (i.e. pannus or thrombus). Therefore, patients may be denied appropriate (surgical) treatment. Our results are in contrast to the findings of Boas and Fleischmann [15]. As stated above, this may be due to the size and composition of the metal object studied, and, probably, to a different algorithm used in their experiments. However, we preferred commercially available techniques in order to test the effectiveness of readily applicable algorithms for PHV scanning.

Recent in vitro work demonstrated that prospectively triggered acquisition on a 256 slice MSCT system generally reduces PHV-related artifacts and image noise substantially compared to retrospectively ECG-gated acquisition at different heart rates [20]. However, further studies are required to evaluate the clinical feasibility of low-dose PHV MSCT protocols that combine prospectively triggered acquisition with iterative reconstruction.

Our study has several limitations. First, we used controlled in vitro conditions which precluded any leaflet motion or annular motion. Motion of high density objects is another factor which may considerably increase the PHV-related artifacts [11]. In earlier work, we demonstrated important variation of artifacts due to leaflet motion [8]. Clinical studies must be undertaken to confirm our in vitro findings. Second, image acquisition was not done with ECG-gating. Although we approximated the exposure parameters to reflect as accurately as possible a cardiac ECG-gated acquisition, the difference between a standard filter and a detailed cardiac filter may affect the amount of image noise and magnitude of artifacts as well as reproducibility of the results in a clinical setting. However, all scans in the current study used the same reconstruction kernel and thus comparison of the different scan and reconstruction protocols is not likely to be affected. Third, our experiments were performed on a 64 slice MSCT system. Currently available higher end MSCT systems (256 slice or more) allow cardiac MSCT imaging with a faster gantry rotation and a higher temporal resolution. Increased temporal resolution may reduce PHV-related artifacts owing to cardiac and/or annular movement, and therefore may improve MSCT image quality. Additional research has to be conducted to assess the differences in PHV-related artifacts and image quality between 64-slice and higher end MSCT systems. Fourth, we used only two PHVs. We chose the St Jude and Medtronic Hall valves because these valves are the most commonly implanted bileaflet and tilting disc valves respectively. In clinical reports, MSCT has been found of additional value for the detection of the cause of dysfunction for both valves [1, 2, 21]. Other commonly implanted PHVs are well visualized by MSCT [4].

In conclusion, our in vitro results suggest that optimizing image quality of PHV with respect to the radiopaque components can be achieved at equal dose with higher tube voltage and to lesser extent with iterative reconstruction. MARF in its current form is very effective in reducing artifacts but the induced interpolation artifacts currently limit its use in clinical PHV imaging.
